# AI-Based Information Systems

**DOI:** 10.1007/s12599-020-00675-8

**Published:** 2020-12-01

**Authors:** Peter Buxmann, Thomas Hess, Jason Bennett Thatcher

**Affiliations:** 1 Wirtschaftsinformatik | Software and Digital Business Group, TU Darmstadt, Darmstadt, Germany; 2 Institut für Wirtschaftsinformatik und Neue Medien, Ludwig-Maximilians-Universität München, Munich, Germany; 3 Department of Management Information Systems, Temple University, Philadelphia, PA USA

## Introduction

Artificial intelligence (AI) is about to bring fundamental changes in our society and economy, touching on how organizations make decisions, deliver services, and evaluate opportunities. Given the breadth of their potential reach across companies of different sizes and in different industries, Erik Brynjolfsson and Andrew McAfee of MIT even speak of AI as “the most important general-purpose technology of our era” (Brynjolfsson and McAfee 2017, p. 2). Today, AI applications in most of the cases are based upon machine learning algorithms, whereby supervised learning, in particular, has become established in practice.

Consistent with this optimistic view, leaders in practice predict the widespread use of AI technologies. Forbes, for example, conducted a study among more than 300 executives. 95 percent of the Forbes study’s participants believe that AI will play an essential role in their companies in the future (Forbes Insights Team 2018). The McKinsey Global Institute (MGI) study predicts that AI’s application in companies will result in a global value-added contribution of USD 13 trillion by 2030 (Bughin et al. 2018).

AI is already being woven into common applications (Buxmann and Schmidt 2021). For example, AI applications that use machine learning algorithms are used to enable essential firm activities, such as analysis of financial credits, to determine the status of production machines, to support essential services, such as law enforcement, and to protect personal data through cybersecurity. Moreover, AI’s application in health care research – e.g., identifying possible treatment plans or use in drug discovery – have assumed even greater importance as scientists search for treatment and vaccinations in COVID-19-times.

From an economic perspective, AI holds the potential to help people, businesses, and governments to lower costs of service delivery and speed up the time required to make decisions. In many cases, algorithms also make faster, more systematic, evidence-based decisions than humans. On the other hand, costs and speed are not the only considerations relevant to decisions. There is a need for a broader conversation about the ethical aspects of decision-making and using AI to make decisions that affect people’s lives. These concerns about the potential risks posed to fairness, non-discrimination, transparency and privacy merit attention from policymakers, business leaders, and academic scientists.

Of course, no one can tell for sure whether these optimistic and pessimistic forecasts will come true. However, such forecasts command attention, suggesting that rather than a passing fashion, AI will be woven into organizations and society in the years to come.

## The Special Issue

In this special issue, we set out to stimulate a conversation about how Artificial Intelligence, a long-established subfield of computer science, is going to shape organizations of the future. We explicitly asked authors to consider if AI and its sub-disciplines such as natural language processing, deep learning algorithms, pattern recognition, knowledge-based systems, or robotics would change organizations. By directing scholars’ attention to the economic and organizational perspective of AI and machine learning, we hoped to gather insights into topics such as organizational AI readiness, the influence of virtual assistants, and aspects of fairness and acceptance of AI.

We received 16 manuscripts that provide a comprehensive coverage of various theories, methods, and contexts. After a developmental peer-review process, four papers remained. We are delighted to include these four papers in our issue.

In the first paper of the special issue, Jöhnk et al. (2021) develop a conceptualization of organizational AI readiness that guides organizations in the AI adoption process. The authors conduct a qualitative study with a total of 25 interviews with AI experts and compare their findings with literature and practitioner studies. In total, they identified 18 relevant AI readiness factors with 58 indicators. Moreover, they structured their results into five categories: Strategic alignment, resources, knowledge, culture, and data. These factors and indicators aim to support organizations in determining their AI readiness level, identify gaps, and make more informed AI adoption decisions.

The paper by Mirbabaie et al. (2021) examines Virtual Assistants (VAs)’ impact on individuals and teams. They use social identity theory to describe the identification of employees with team members and the continued existence of group identity. Using a laboratory experiment, they compared two groups in solving a task. One group was assisted by a VA, while the other was supported by a person. The main result of this study is that the relationship between social identification with (virtual) team members and expanding the self through technology such as VAs is not contradictory but rather complementary.

Köchling et al. (2021) discuss the issue of bias and algorithmic fairness in the context of algorithmic decision-making for recruitment. An exploratory data analysis is carried out on a data set containing 10.000 15-s video clips to examine personality traits and invitations for job interviews. The findings encompass two main insights: First, the use of algorithmic decision-making does not eliminate the threat of implicit biases and discrimination. Second, underrepresentation in a data set concerning gender and ethnicity in the training data can cause unpredictable classifications. For example, the selection of interview representatives in the recruitment process is biased towards specific groups.

Finally, the paper of Berger et al. (2021) deals with algorithmic aversion in the context of AI. Here, an online experiment examines how participants react to advice from a human advisor instead of an algorithm’s advice. The experiment also investigates the potential effect of participants becoming familiar with the algorithmic advisor during the experiment and that advisor demonstrates its ability to learn. Overall, the experiment indicates that familiarity with an erring algorithmic advisor reduces decision makers’ reliance on this advisor and shows that demonstrating an algorithm’s ability to learn can offset this effect over time.

The special issue also includes an interview with Karl-Heinz Streibich on AI. The president of acatech explains from a practical point of view what challenges companies face when adopting AI and discusses AI as the next evolutionary step in using IT and digital systems.

## What’s Next?

We hope that this special issue provides a starting point for scholars interested in understanding the implications of AI for people, organizations, and society. There remain many interesting topics that our authors’ work suggests merit attention from economic and organizational scholars. For example:

- *AI, CIOs, and Firm Strategy* The work of Jöhnk et al. (2021) points to a need to examine strategic issues tied to AI’s deployment in organizations. While we know how CIOs manage strategic and maintenance issues concerning technology, we know little about how they go about marshalling resources necessary to develop and sustain technologies that can fundamentally change how firms make decisions and deliver services. Key lingering questions include: How do CIOs align AI deployment with firm strategy? Will CIOs become more prominent in firm governance as they acquire responsibility for supporting strategic AI decisions? What role will boards of directors play in shaping AI strategy? While AI readiness constitutes a first step towards understanding strategic issues because AI represents a fundamental change in how technology impacts business, we may need to revisit our present understanding of how CIOs shape IT strategy as well as ask if new forms of governance will be required to effectively deploy and maintain AI in organizations.
- *AI, Identity, and Sociotechnical Systems* Mirbabaie et al. (2021) build on the growing stream of work on individuals and IT identity and point to a need to progress from considering the individual to considering the identity of groups and the technology itself. While we know individuals possess IT identity, will AI learn to support the quirks and habits of teams? Will it enable the formation of unique identities relative to the systems that support them? If so, how will that change the interaction of not only individuals within groups but also that of groups vis-a-vis the broader sociotechnical context in which they work? Further, as AI grows more sophisticated, autonomous, and capable of double-loop learning, how will the “identity” manifest in the algorithms shape when we interact with them? And integrate them into the firm processes? This work on virtual assistants constitutes only a first step towards a broader understanding of the implications of using AI to support teams and to integrate them with broader sociotechnical systems.
- *AI, Bias, and Data* Köchling et al. (2021) underscore that AI is prone to the biases imbued by rules and data provided by their human designers. While the findings illustrate how biases in algorithms and data can result in adverse impacts on different groups of people, their implications are further reaching for designing and understanding AI. This work suggests a need for thoughtful, introspective research that examines how to collect data that accurately depicts the “ground truth” of organizational and social life, as we design but also maintain algorithms targeted at supporting fairness and equity in organizations and society. How can we know data represents the full set of factors relevant to desired outcomes? How can we ensure people trust the AI and do so sufficiently to continue sharing information with it? What role will privacy play? And security? In determining to which extent are people willing to share data needed to train AI and sustain them? How can we train AI to detect biases? Or what about adverse impacts for the people they serve?
- *AI, the Uncanny Valley, and the Singularity* Berger et al. (2021) demonstrate that people trust AI that demonstrates a capacity to improve. While much has been made of anthropomorphism and the uncanny valley, there remains a lot to learn about how to design the manner in which we interact with AI, particularly, AI that will soon be able to emulate humans. Their work suggests a need for careful investigation of not only how we present the AI (e.g., the interface) but also of how we educate users about the algorithms that drive the AI, the relationship from data fed into the AI to outcomes and to users’ affective responses to the support offered by AI. How will users respond to increasingly human-like systems? Will a greater understanding of algorithms result in more trust? And information sharing? Or will it undercut beliefs about security and exacerbate fears about privacy? Should designers avoid the uncanny valley or embrace the “singularity”? And should they design systems to be partners with users?
- *AI and Ethics* Collectively, these papers suggest a need to actively question the implications of how we build the algorithms, gather data to train, and apply AI to solve problems in organizations and society. These papers hint at the necessity to investigate ethical questions, such as: How does the design of AI change the decisions that we make? Do we fully inform users of the scope of AI application in the sociotechnical systems they live in? Or do we apply AI like electricity – as a utility? And without comment?
  In our minds, the papers in this special issue evoke questions about user data, its sources, and application. In how far are designers obliged to explain the implications of users’ training data contributions? And what about the potential applications of their data to solving problems? Or enabling decisions in AI-enabled information systems? And given applications of data will certainly evolve, what ethical obligations do organizations and designers have to update contributors about new uses of data they shared? These questions about data are particularly salient as GDPR becomes infused with the intellectual framework of our society – and is not in the culture of other societies.
  Since we first crafted the call for contributions to the special issue and when reflecting on these papers, we found ourselves asking even bigger questions about ethics and AI that merit attention in future work in BISE and other rigorous academic outlets – what limits should we place on AI in society? Just because we can design systems to make decisions, should we do so? What kind of problems should unsupervised AI be applied to? What kind of decisions should remain at least semi-supervised by humans? As we allocate decisions between humans and AI, what role should ethics play in the allocation of responsibilities between people and machines? How can we ensure that AI is applied in a way that results in pareto efficient outcomes for people and society?

These are just a few of the questions and opportunities that this special issue presents for future research on artificial intelligence. There are many more that we lacked room to integrate into this special issue, e.g. about the ethics of design of designers, and of how governments use AI. We look forward to seeing how the next wave of AI researchers address these questions as they seek to build a better society through the judicious integration of AI into individuals’ lives and the organizations in which they work.

## Special Thanks

We would like to offer our sincere thanks to all authors and reviewers who have contributed to the research presented in this special issue. Special thanks go to the Business & Information Systems Engineering team, without whom the special issue would not have been possible, and to Anne Zöll from Technical University of Darmstadt for her organizational support during the publication process. We are very pleased to present the methods, concepts, and results of this special issue to researchers and practitioners. We hope that these contributions will enhance the field of AI-based Information Systems.
